# Therapeutic efficacy and artemisinin resistance in northern Myanmar: evidence from in vivo and molecular marker studies

**DOI:** 10.1186/s12936-017-1775-2

**Published:** 2017-04-07

**Authors:** Moe Kyaw Myint, Charlotte Rasmussen, Aung Thi, Dorina Bustos, Pascal Ringwald, Khin Lin

**Affiliations:** 1 Department of Medical Research (Pyin Oo Lwin Branch), Ministry of Health and Sports, Pyin Oo Lwin, 05062 Myanmar; 2 World Health Organization, 20 Avenue Appia, 1211 Geneva 27, Switzerland; 3 National Malaria Control Programme, Ministry of Health and Sports, Nay Pyi Taw, Myanmar; 4 World Health Organization, Bangkok, Thailand

**Keywords:** Myanmar, Treatment efficacy, *Plasmodium falciparum*, Molecular markers, *k13*

## Abstract

**Background:**

In Myanmar, three types of artemisinin-based combination therapy (ACT) are recommended as first-line treatment of uncomplicated falciparum malaria: artemether–lumefantrine (AL), artesunate–mefloquine (AS + MQ), and dihydroartemisinin–piperaquine (DP). Resistance to both artemisinins and ACT partner drugs has been reported from the Greater Mekong Sub-region, and regular efficacy monitoring of the recommended ACT is conducted in Myanmar. This paper reports on results from studies to monitor the efficacy of the three forms of ACT in sentinel sites in northern Myanmar, and investigations of mutations in the *Kelch13 (k13)* propeller domain.

**Methods:**

Seven therapeutic efficacy studies were conducted in 2011–12 and 2014 in three sentinel sites in Myanmar (Tamu, Muse, Tabeikkyin). Three studies were done for the evaluation of AL (204 patients), two studies for AS + MQ (119 patients) and two studies for DP (147 patients). These studies were done according to 2009 standard WHO protocol. Polymorphisms in the *k13* propeller domain were examined in dried blood spots collected on day 0. The primary endpoint was adequate clinical and parasitological response (ACPR) on day 28 for AL and on day 42 for DP and AS + MQ, corrected to exclude re-infection using polymerase chain reaction (PCR) genotyping. Safety data were collected through self-reporting.

**Results:**

PCR-corrected ACPR was 97.2–100% for AL, 98.6–100% for AS + MQ and 100% for DP across the study sites and years. All studies found a prevalence of *k13* mutations (>440) above 23% in the day-0 samples. The F446I mutation was the most common mutation, making up 66.0% of the mutations found. Seven out of nine day-3 positive patients were infected with *k13* wild type parasites. The remaining two cases with day-3 parasitaemia had the P574L mutation.

**Conclusions:**

The efficacy of AL, AS + MQ and DP remains high in northern Myanmar despite widespread evidence of *k13* mutations associated with delayed parasite clearance. This study showed that already in 2012 there was a high frequency of *k13* mutations in Myanmar on the border with India. The high efficacy of the recommended ACT gives confidence in the continued recommendation of the use of these treatments in Myanmar.

*Trial registration numbers* ACTRN12611001245987 (registered 06-12-2011) and ACTRN12614000216617 (registered 28-02-2014)

## Background

In 2014, Myanmar reported 152,195 malaria cases, 72% of them due to *Plasmodium falciparum.* Overall, this is a 74% reduction from the number of cases reported in 2010 [1]. A key factor in this significant reduction of malaria in Myanmar has been expanded access to artemisinin-based combination therapy (ACT). Currently, there are three ACT recommended as first-line treatment for *P.* *falciparum*: artemether–lumefantrine (AL), artesunate–mefloquine (AS + MQ) and dihydroartemisinin–piperaquine (DP) [2]. A decline in ACT efficacy has been seen in other countries in the Greater Mekong Sub-region (GMS), and could pose a threat to continued progress towards malaria elimination [3].

Myanmar is the only country in the GMS with more than one recommended first-line ACT in a given geographical area. While AL is the treatment that is most frequently used in the public sector and by most international non-governmental organizations, the other ACT are available in the private sector, making monitoring of all three recommended ACT a necessity. Therapeutic efficacy studies (TES) for anti-malarial medicine are the gold standard, providing the information needed to guide treatment policies. Monitoring of molecular markers for resistance can provide supplementary information and is helping to improve the surveillance of resistance.

All ACT contain an artemisinin and a partner drug. Resistance to artemisinin was first identified in clinical studies in 2006 close to the Cambodia–Thailand border. However, retrospective analysis of molecular markers indicates that artemisinin resistance likely emerged as early as 2001. Artemisinin resistance is defined as delayed parasite clearance following treatment with an artesunate monotherapy or with an ACT. In TES, this is measured by the proportion of patients found positive on day 3 after treatment [4].

Artemisinin resistance has been shown to be associated with point mutations in the propeller region of the *P. falciparum Kelch13* (*k13*) gene [5]. At present, 108 different non-synonymous *k13* mutations have been reported, and these different mutations can have varying effect on the clearance genotype. Studies have shown a clear distinction between the *k13* mutations that are most frequent in eastern GMS (Cambodia, Lao PDR, Vietnam) and western GMS (China, Myanmar, Thailand) [4].

In Myanmar, delayed parasite clearance in patients treated with an ACT was first seen in 2009 in southern Myanmar [6]. Recent studies have shown high prevalence of *k13* mutations extending through much of Myanmar [7, 8]. However, artemisinin resistance and delayed parasite clearance will not necessarily lead to treatment failure as TES of ACT for treatment of falciparum malaria have continued to show high efficacy. In the GMS, high treatment failure rates following treatment with an ACT have mostly been observed where concomitant resistance to the partner drug exists [4].

This paper reports on the results of seven therapeutic ACT efficacy studies of the three recommended ACT in 2011–12 and 2014 in northern Myanmar, and results from investigations of mutations in the *k13* propeller domain.

## Methods

### Study design and sites

**Fig. 1:**
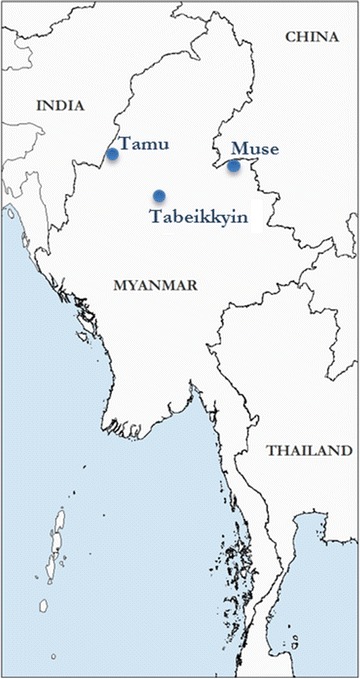
Map showing location of study sites

Two studies were conducted in September 2011–September 2012 to evaluate AL and AS + MQ in Muse in northern Myanmar, close to the border with China. In Tamu, close to the Indian border, two studies were done in June–October 2012 to evaluate AL and AS + MQ. Three additional studies were done in May–November 2014: two in Tabeikkyin to evaluate AL and DP, and one in Tamu to evaluate DP. Location of the sites is shown in Fig. 1.

The study design was an open-label, one-arm, prospective evaluation of clinical and parasitological responses to directly observed treatment for uncomplicated malaria. The WHO protocol for monitoring therapeutic efficacy was used [9]. In the sites where two different ACT were assessed, the treatments were studied sequentially so that recruitment of patients for the assessment of one ACT was completed before patients were recruited for assessment of a second ACT.

Estimation of the target sample size in each study was based on an assumption of treatment failure of 5%, with a 95% confidence level and precision of 5%. Allowing for a 10% loss to follow-up, the target sample size in each study was 80 cases. Ethical approvals were obtained from the Ethics Review Committee of the Department of Medical Research, Yangon, Myanmar, and the WHO Ethics Review Committee, Geneva, Switzerland. The studies were registered at Australian New Zealand Clinical Trials Registry [10].

### Study population

Cases were enrolled after obtaining a patient’s, or parent’s/guardian’s written consent. Eligible patients were aged six years and above (excluding females 12–17 years) with microscopically confirmed, uncomplicated *P. falciparum* mono-infection, a parasite density of 500–100,000 asexual parasites/μl, and a body temperature ≥37.5 °C, or a history of fever during the previous 24 h. Pregnant women were excluded from the study, as were patients with signs of severe malaria or with febrile conditions due to diseases other than malaria.

### Treatment

AL (Coartem^®,^ Novartis, Switzerland) was administered at a target dose of 1.3/8 mg/kg twice daily for 3 days. For treatment with AS + MQ, artesunate (Guilin Pharmaceutical Ltd, China) was given at a target dose of 4 mg/kg once a day for 3 days. Mefloquine (Mepha Ltd, Switzerland) was given at a target dose of 15 mg/kg once on the first day followed by 10 mg/kg on the second day. DP (Duo-Cotecxin^®^, Zhejiang Holley Nanhu Pharmaceutical Ltd, China) was given at a target dose of 2–2.4/16–19.2 mg/kg once a day for 3 days. Quality-controlled medications for the studies were provided by WHO Global Malaria Programme.

### Patient follow-up

Patients received medications under direct observation on the first 3 days. Clinical and parasitological follow-up was done on days 0, 1, 2, 3, 7, 14, 21, and 28 for AL, and additionally on days 35 and 42 for AS + MQ and DP. Any adverse events reported by patients were recorded at each visit. Monitoring included axillary temperature, parasitaemia and gametocytaemia.

### Laboratory methods

Thick and thin blood films were obtained from each patient at screening. Blood films were also obtained on days 1, 2, 3, 7, 14, 21, 28, (and 35 and 42 for AS + MQ or DP), and any other day if the patient returned spontaneously and parasitological re-assessment was needed. The blood smears were stained with fresh Giemsa, and examined at a magnification of 1000× to identify parasite species and determine parasite density. Asexual parasitaemia was determined from Giemsa-stained thick blood smears against the number of parasites per 200 white blood cells on day 0, based on an assumed density of 6000 white blood cells per µl of blood. Gametocytes were similarly enumerated. A blood smear was declared negative after examination of 1000 white blood cells [9]. Quality control of microscopy for all studies was done by an external consultant from the Research Institute for Tropical Medicine in the Philippines, a WHO Collaborating Centre for malaria diagnosis.

Dried blood spots were obtained for polymerase-chain reaction (PCR) at enrolment (day 0) and on follow-up days 7, 14, 21, and 28 (and 35 and 42 for AS + MQ or DP). PCR genotyping was performed on paired dried blood spots in the case of parasitaemia detected on or after day 7 to distinguish between recrudescence and re-infection. This PCR genotyping determined polymorphisms in merozoite surface protein-l (*msp*-*1*), merozoite surface protein-2 (*msp*-*2*) and glutamate-rich protein (*glurp*) polymorphism, as per WHO methods [9]. The PCR testing was done at the laboratory of Department of Medical Research, Yangon, Myanmar.

Polymorphisms in the *k13* propeller domain were examined in the dried blood spots collected on day 0. The method used was a nested PCR protocol followed by Sanger sequencing using primers specific to *P. falciparum*. The amplicon used for sequencing covered 740 bp, which included the *k13* propeller domain [11]. The sequencing was done at Faculty of Tropical Medicine, Mahidol University, Bangkok, Thailand, and at the Malaria Molecular Epidemiology Unit, Institut Pasteur, Phnom Penh, Cambodia.

### Outcome evaluation and analysis

Treatment outcomes were classified on the basis of an assessment of the parasitological and clinical outcome of anti-malarial treatment according to WHO guidelines. All patients were classified as having early treatment failure (ETF), late clinical failure (LCF), late parasitological failure (LPF), or an adequate clinical and parasitological response (ACPR) [9].

The primary outcome measure was PCR-corrected ACPR. The patients were excluded from the analysis if PCR results were not available to check for re-infection. If PCR results suggested re-infection, then the patient was excluded from the pre-protocol analysis.

Information on ACT-related side effects was collected through self-reporting and recorded in the case reporting forms. EpiData software was used for double data entry (and validation) and analysis (version 3.1 for entry and version 2.2.2.182 for analysis, EpiData Association, Odense, Denmark).

## Results

**Fig. 2:**
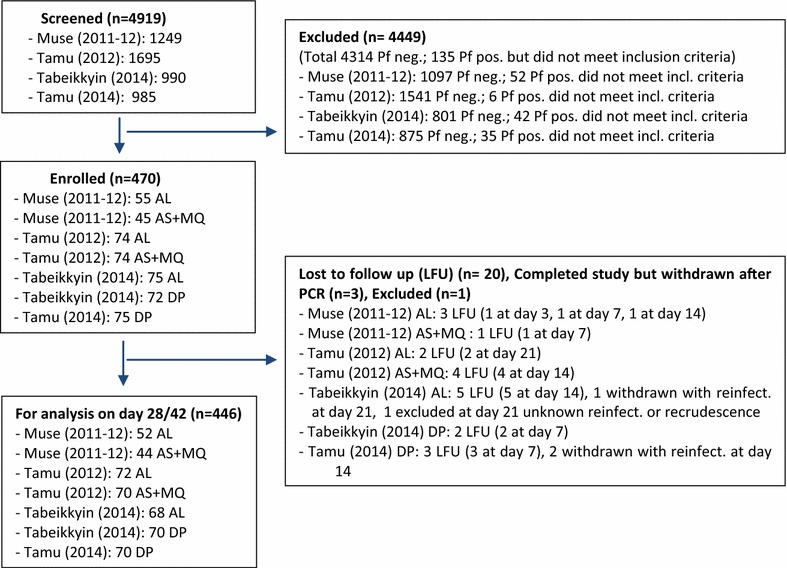
Participant flow chart

Seven studies were conducted in three sentinel sites in Myanmar in 2011–2012 and 2014 to assess the efficacy of AL (three studies), AS + MQ (two studies) and DP (two studies). A total of 4919 patients were screened and 470 enrolled. Of these, 20 patients were lost to follow-up before day 28/42, three patients were withdrawn due to PCR-confirmed re-infection, one patient was excluded from the analysis with parasitaemia without PCR being able to determine re-infection or recrudescence, leaving 446 patients for evaluation at day 28/42 (Fig. 2).

### Baseline characteristics of enrolled patients

The demographic and clinical characteristics of the study population in each site is shown in Table 1. The majority of the enrolled patients in all studies were males (66.7–91.1%). The mean age in the studies was between 22.8 and 39 years with the majority of the patients being above 15 years (80–100%). The geometric mean of parasitaemia at admission in the seven studies was 3210–5199 asexual parasites/μl.

**Table 1:** Baseline characteristics of patients by study

Site	Year	Drug	Enrolled n	Males n (%)	Age		Weight mean kgs (range)	Parasitaemia (/ml) geometric mean (range)
					Mean years (range)	>15 years n (%)		
Muse	2011–12	AL	55	45 (81.8)	22.8 (5–56)	44 (80)	56.9 (30–70)	3745 (1002–76,421)
Muse	2011–12	AS + MQ	45	41 (91.1)	29.6 (16–65)	45 (100)	59.4 (50–65)	3210 (1000–54,436)
Tamu	2012	AL	74	64 (86.5)	33.2 (11–60)	71 (95.9)	55.8 (30–70)	3494 (1002–31,492)
Tamu	2012	AS + MQ	74	62 (83.8)	36.6 (13–64)	73 (98.6)	58 (40–75)	3774 (1081–15,310)
Tabeikkyin	2014	AL	75	52 (69.3)	36.6 (8–60)	74 (98.7)	52.9 (30–65)	4251 (1524–12,244)
Tabeikkyin	2014	DP	72	60 (83.3)	35.6 (12–65)	71 (98.6)	55 (32–65)	4408 (1063–16,424)
Tamu	2014	DP	75	50 (66.7)	39 (10–74)	72 (96.0)	56.9 (32–75)	5199 (2556–14,664)

### Adverse events

Overall, 27% of all study participants reported one or more side effects (Table 2). With AS + MQ, 33% reported side effects, for AL 26% reported side effects, and for DP 22% reported side effects. The most frequent side effect by type of ACT was insomnia (10.9%) with AS + MQ, dizziness (9.3%) with AL, and vomiting (6.8%) with DP. No serious adverse events were reported.

**Table 2:** Adverse events reported by treatment

	Total (n = 470)	Artemether + lumefantrine (n = 204)	Artesunate + mefloquine (n = 119)	Dihydroartemisinin + piperaquine (n = 147)
	n (%)	n (%)	n (%)	n (%)
Number of adverse events per patient				
0	345 (73)	150 (74)	80 (67)	115 (78)
1	60 (13)	25 (12)	16 (13)	19 (13)
2	64 (14)	29 (14)	22 (19)	13 (9)
3	1 (<1)	0	1 (<1)	0
Most frequent type of adverse event reported				
Dizziness	30 (6.4)	19 (9.3)	9 (7.6)	2 (1.4)
Nausea	29 (6.2)	13 (6.4)	8 (6.7)	8 (5.4)
Vomiting	27 (5.7)	8 (3.9)	9 (7.6)	10 (6.8)
Loss of appetite	19 (4.0)	9 (4.4)	3 (2.5)	7 (4.8)
Insomnia	15 (3.2)	2 (1.0)	13 (10.9)	0
Palpitation	14 (3.0)	11 (5.4)	2 (1.7)	1 (0.7)
Diarrhoea	13 (2.8)	3 (1.5)	3 (2.5)	7 (4.8)
Abdominal pain	10 (2.1)	3 (1.5)	7 (5.9)	0
Asthaenia	10 (2.1)	1 (0.5)	4 (3.4)	5 (3.4)
Headache	6 (1.3)	6 (2.9)	0	0
Cough	6 (1.3)	2 (1.0)	0	4 (2.7)
Joint/muscle pain	5 (1.1)	3 (1.5)	1 (0.8)	1 (0.7)
Skin rash	3 (0.6)	2 (1.0)	1 (0.8)	0
Nightmare	2 (0.4)	0	2 (1.7)	0

### Clinical and parasitological responses

Cases positive on day 3 were identified in three of the seven studies (Table 3). In total, only nine of the 470 enrolled patients were found to be positive on day 3. All the day-3 positive cases were followed up to day 28/42, and all had ACPR. The highest number of day-3 positive cases was in the 2011–12 study in Muse monitoring efficacy of AL: 4 of 55 (7.3%) patients were still positive at day 3. Of the nine day-3 positive cases, eight were identified in studies for AL efficacy. The geometric mean of day 0 parasitaemia (/μl) among the nine day-3 positive cases was 5962 (range 1079–31,492). At day 3, the geometric mean of parasitaemia (/μl) among the positive cases was 69 (range 23–131).

**Table 3:** Parasitological responses by study

Site	Year	Drug	Enrolled n	Day 3 Pos. n (%)	Non-PCR corrected			PCR corrected			
					Excl./loss	LPF	ACPR n (%)	Excl./loss	LPF	ACPR n (%)	KM cure rate % (95% CI)
Muse	2011–12	AL	55	4 (7.3)	3	0	52 (100)	3	0	52 (100)	100 (n/a)
Muse	2011–12	AS + MQ	45	1 (2.2)	1	0	44 (100)	1	0	44 (100)	100 (n/a)
Tamu	2012	AL	74	4 (5.4)	2	2	70 (97.2)	2	2	70 (97.2)	97.3 (89.6–99.3)
Tamu	2012	AS + MQ	74	0 (0)	4	1	69 (98.6)	4	1	69 (98.6)	98.6 (90.3–99.8)
Tabeikkyin	2014	AL	75	0 (0)	5	2	68 (97.1)	7	0	68 (100)	100 (n/a)
Tabeikkyin	2014	DP	72	0 (0)	2	0	70 (100)	2	0	70 (100)	100 (n/a)
Tamu	2014	DP	75	0 (0)	3	2	70 (97.2)	5	0	70 (100)	100 (n/a)

No early treatment or late clinical failures recorded

*LPF* late parasitological failure, *ACPR* adequate clinical and parasitological response, *KM* Kaplan-Maier, *CI* confidence interval

Table 3 shows parasitological responses for the different studies. The proportion of cases with ACPR was 97.1–100% before PCR correction, and 97.2–100% after PCR correction. Three patients were classified as treatment failures after PCR correction and the exclusion of patients with re-infections. All three cases were identified with recrudescence on day 21 in the 2012 studies in Tamu: two of them in the study for AL, and the third in the AS + MQ study. None of the three patients with treatment failures was identified as having parasitaemia on day 3.

### *k13* Genotyping

The *k13* gene was successfully sequenced in 288 (61.3%) of the 470 day-0 samples. The main reason for failure to do sequencing was that for some patients too little blood was collected on the filter papers. Among the sequenced samples, the prevalence of *k13* mutations (>440) was 33.7% (97/288) (Table 4). The F446I mutation was the most common mutation, making up 66.0% (64/97) of the mutations being present in 22.2% of the samples. The highest prevalence of *k13* mutations was in Thabeikkyin in 2014 (49.2%).

**Table 4:** Prevalence of *k13* mutations by study

*k13* genotype	Muse 2011–12			Tamu 2012			Tabeikkyin 2014			Tamu 2014	Grand total n (%)
	AL n	AS + MQ n	Total n (%)	AL n	AS + MQ n	Total n (%)	AL n	DP n	Total n (%)	DP n (%)	
F446I	1	8	9 (12.7)	19	6	25 (25.8)	10	6	16 (25.4)	14 (24.6)	64 (22.2)
N458Y	0	0	0 (0)	0	0	0 (0)	3	4	7 (11.1)	0 (0)	7 (2.4)
S459L	0	0	0 (0)	0	0	0 (0)	0	1	1 (1.6)	0 (0)	1 (0.3)
P553L	0	0	0 (0)	0	1	1 (1)	1	0	1 (1.6)	0 (0)	2 (0.7)
R561H	1	1	2 (2.8)	0	0	0 (0)	3	3	6 (9.5)	7 (12.3)	15 (5.2)
P574L	5	0	5 (7)	0	1	1 (1)	0	0	0 (0)	0 (0)	6 (2.1)
A676D	0	0	0 (0)	0	1	1 (1)	0	0	0 (0)	0 (0)	1 (0.3)
P701R	1	0	1 (1.4)	0	0	0 (0)	0	0	0 (0)	0 (0)	1 (0.3)
Total *k13* mutations	8	9	17 (23.9)	19	9	28 (28.9)	17	14	31 (49.2)	21 (36.8)	97 (33.7)
Wild type	38	16	54 (76.1)	41	28	69 (71.1)	16	16	32 (50.8)	36 (63.2)	191 (66.3)
Total cases genotyped	46	25	71	60	37	97	33	30	63	57	288

For the three cases with treatment failure, *k13* sequencing was not possible. Sequencing showed that *k13* genotypes in seven of the nine cases with parasitaemia on day 3 were *k13* wild type parasites. The remaining two cases with day-3 parasitaemia had the P574L mutation. While the numbers are small, there is evidence (p < 0.05 using a Fisher’s exact test) of significant differences in the proportion of day-3 positive cases by genotype.

## Discussion

This paper reports on the therapeutic efficacy of all three recommended first-line ACT from sentinel sites in northern Myanmar. The efficacy of all ACT in the studies were high (>97%) in both PCR uncorrected and corrected analyses, meeting the WHO recommendation that cure rates for falciparum malaria should be at least 90% [9]. These results are similar to results found in other studies in northern Myanmar [12, 13].

A total of eight different *k13* mutations (position >440) were recorded in the studies. Artemisinin resistance was first identified in southern Myanmar close to the border with Thailand where the most extensive data are from. In the 2012 study in Tamu on the Myanmar-India border, *k13* mutations were found in 28.9% of the samples, mainly the F446I mutation (25 of 28 *k13* mutations). These are the earliest data documenting *k13* mutations in this part of Myanmar.

Data from the Indian side of the border show that AL is highly efficacious (Mishra and Valecha, pers. comm.). AL was introduced as first-line treatment in northeastern India after increasing treatment failures with artesunate + sulfadoxine–pyrimethamine (AS + SP) due to resistance to SP. Data from 2010–2013 showed very low prevalence of *k13* mutations in India. Four non-synonymous *k13* mutations were identified among 384 samples but only at very low frequencies (0.26%, 1/384). Of these four mutations, three (G533A, R561H and A578S) were detected in the northeastern states [14]. Of these three mutations, only the R561H were identified in Tamu and only in the 2014 study.

The most common mutation in all the Myanmar study sites was the F446I. In Tamu in 2012, the prevalence of F446I was 25.8%. This remained nearly constant with the prevalence of F446I in Tamu in 2014 at 24.6%. One mutation in Tamu not found in 2012, but found in seven patients (12.3%) in 2014 was the R561H mutation. In Tabeikkyin in 2014, the N458Y mutation was identified in seven patients (11.1%). Both the F446I and the R561H mutations have been reported in other studies from northern Myanmar [7, 12, 15]. The N458Y mutation has previously been identified in southern and central Myanmar [7]. Two highest prevalent mutations found in Muse in Myanmar–China border in 2011–2012 were the F446I and P574L. This finding is similar to other study done in that border area [15]. The C580Y mutation was not found in the samples. However, other study done in southern part of Myanmar found C580Y mutation in Kayin State, Myanmar-Thailand Border [7]. This mutation is common in the eastern part of the GMS, becoming increasingly dominant among *k13* mutations in Cambodia [16].

The prevalence of molecular markers for artemisinin resistance found in the studies has not resulted in a high prevalence of day-3 positivity, or a fall in efficacy of the ACT. The majority (7/9) of the day-3 positive cases were *k13* wild type parasites. The overall percentage of patients positive on day 3 is only 3.1%. However, among patients identified as having the P574L mutation, 33% (2/6) was found to have day-3 parasitaemia. Despite the low number of patients, there is evidence of significant differences (p < 0.05) in the proportion of day-3 positive cases by genotype.

The overall low proportion of day-3 positive patients could be for several reasons. The patients had significant lower parasitaemia on day 0 than in other research studies done in the area, which makes it less likely that parasites would be found on day 3, even if there are relatively slow parasite clearance rates. These studies do not allow for estimation of parasite clearance half-life as blood was collected only once a day rather than every 6 or 8 h. Another possible explanation for the low proportion of cases positive on day 3 is the prevalence of *k13* mutation. The F446I mutation may be associated with only an intermediate artemisinin resistance phenotype, not resulting in a slowing of the parasite clearance to the same extent as mutations most prevalent in eastern parts of the GMS [12]. In addition, factors such as immunity and pharmacokinetics may have played a role.

A high efficacy was found for all the three recommended ACT, giving confidence in the continued recommendation of these ACT as appropriate first-line treatments for *P.* *falciparum.* So far studies done in all sentinel site of Myanmar showed high level of efficacy (>90%) to the three recommended ACT. The role of triple ACT for treatment of uncomplicated falciparum malaria can be determined only after the completion of ongoing ACT versus triple ACT studies in Myanmar. Nevertheless, continued monitoring will be needed. While there is no evidence for ACT partner drug resistance resulting in ACT failures in these studies, partner drug resistance is leading to ACT failures in other GMS countries, emphasizing the risks and the need to continue monitoring ACT efficacy.

Reporting of adverse events showed that among patients receiving AS + MQ, there was a slightly higher proportion of patients reporting one or more adverse events. This could affect the effectiveness of the treatment, as a higher proportion of patients on AS + MQ may choose to discontinue treatment before completion of the full three-day treatment.

## Conclusion

All the three recommended ACT in Myanmar have been shown to be highly efficacious despite widespread evidence of *k13* mutations associated with delayed parasite clearance after treatment with an artemisinin. This study showed that already in 2012 there was a high frequency of *k13* mutations in Myanmar on the border with India. The high efficacy of the recommended ACT gives confidence in the continued recommendation to use these treatments in Myanmar. The threat of resistance to ACT partner drugs resulting in falling ACT efficacy elsewhere in the region highlights the need for continual monitoring of ACT efficacy.
